# A Spectroscopic Methodology to Early Detection of Urinary Tract Infections

**DOI:** 10.3390/s25020400

**Published:** 2025-01-11

**Authors:** Ana F. N. S. Mendes, Nuno Matela, João M. P. Coelho, Joaquim T. Marquês

**Affiliations:** 1 Instituto de Biofísica e Engenharia Biomédica (IBEB), Faculdade de Ciências, Universidade de Lisboa, Campo Grande, 1749-016 Lisboa, Portugal; fc49806@alunos.fc.ul.pt (A.F.N.S.M.); nmatela@ciencias.ulisboa.pt (N.M.); jmcoelho@ciencias.ulisboa.pt (J.M.P.C.); 2 Centro de Química Estrutural, Institute of Molecular Sciences, Departamento de Química e Bioquímica, Faculdade de Ciências, Universidade de Lisboa, Campo Grande, 1749-016 Lisboa, Portugal

**Keywords:** urinary tract infections, urine monitoring, fluorescence spectroscopy, ratiometric measurements, transmittance

## Abstract

Healthcare-associated infections (HAI) are a critical public health problem, with 30 to 40% of infections related to the urinary tract system. These urinary tract infections (UTIs) are considered one of the most common microbial infections in hospital settings and everyday community contexts, where approximately 80% are highly correlated with urinary catheter insertion, i.e., catheter-associated urinary tract infections (CAUTIs). Considering that 15 to 25% of hospitalised patients need to be catheterised during their treatments and most CAUTIs are asymptomatic, it results in a tremendous challenge to provide an early diagnosis of CAUTI and therefore initiate its treatment. The lack of standardised methods as a first step for urine monitoring and early detection of UTIs is the driving force of this work, which aims to explore the potential of absorption and fluorescence spectroscopic methodologies to detect UTIs. Urine samples were used without any previous treatment to target the most straightforward testing protocol possible. In this work, we successfully developed a powerful methodology that combines ratiometric fluorescence spectroscopy measurements and transmittance at 600 nm to distinguish healthy urine from infected urine. The complementary use of fluorescence spectroscopy and transmittance is what makes the new methodology we propose such a powerful approach to monitor urine samples and provide early detection of UTIs since it provides a quantitative analysis of both healthy and infected urine.

## 1. Introduction

Despite the remarkable advances in healthcare over the years, there is still a long way to go regarding healthcare-associated infection (HAI), which is recognised by the World Health Organization (WHO) as a public health problem [1].

This health problem remains one of the leading causes of death worldwide and represents a significant economic burden. WHO has reported the annual financial damage due to HAI in Europe to be EUD ~7 billion, whereas, in the United States, it is USD ~6.5 billion dollars when just considering the direct costs [1].

Urinary Tract Infections (UTIs) are among the most frequent HAIs (about 30 to 40%) and constitute one of the most common microbial infections in hospitals or even in the normal community context [2]. Despite the high prevalence of UTIs, the main challenge is to achieve a timely and accurate diagnosis since UTIs may have many different causative agents.

The main symptoms of UTI are dysuria, i.e., the presence of discomfort and burning sensation in the urethra during micturition and an abnormal increase in its frequency. Other symptoms can also be back pain, fever, cloudiness, dark blood, or bad-smelling urine. However, once these symptoms are identified, it is no longer possible to avoid infection, and the course of action is to proceed with treatment, which can be more or less aggressive, according to the severity of the infection. Thus, the early identification of microorganisms in urine is of pivotal importance in the timely detection of UTI.

Regarding UTIs resulting from HAI, 80% are related to urinary catheterisation, described as catheter-associated urinary tract infections (CAUTI). According to some reports, approximately 15 to 25% of all hospitalised patients have to be catheterised during their hospital treatments [2]. Furthermore, for a single day of catheterisation, the risk of developing UTI is between 1 and 2%; however, for each additional day, this risk increases 10% in women and 3 to 4% in men [3,4]. As a result, after 30 days, it is possible to infer that all patients are likely to report bacteria in their urinary tract, known as bacteriuria [5]. Around 70 to 80% [6,7] of the most complicated UTIs are highly correlated with the insertion of foreign bodies, such as indwelling catheters, which allow the adherence of microorganisms and biofilm formation. These are mostly asymptomatic infections, especially for a catheter utilisation for less than 30 days, making diagnosis extraordinarily difficult and also delayed. In fact, asymptomatic bacteriuria is very common to develop, and approximately 95% of patients have detectable bacteriuria only after two weeks or more when catheterised [8]. There is a correlation between the acquisition of a CAUTI and the mortality rate in intensive care units [9], and the mortality rate due to UTI can be as much as 9% [10]. Thus, there is a considerable risk of a silent worsening of the patient’s state of health during their hospitalisation due to CAUTI, which may culminate even in death.

CAUTI is an important public health problem. Its mitigation would improve patient health and result in a significant decrease in HAI, which, in turn, would lead to economic relief through a reduction in hospital expenses, patient hospitalisation days and additional labour hours of health professionals [6,11].

Urine is a highly complex biological fluid mainly composed of water (≃95%) and containing a low percentage of inorganic salts and soluble metabolic residues [12]. To date, over 3000 compounds have been identified in urine [12,13]. Its composition is highly volatile, and it depends on multiple factors such as gender, age, diet, metabolism, physical activity, and the presence of medications. In addition to these variables, the urine composition from the same patient can also change over time on the same day. However, a sample of urine provides a very detailed report of the patient’s internal physiological status. Many components have already become potential biomarkers for diagnosing and monitoring several diseases, such as prostate cancer [14], bladder cancer [15], and acute kidney injury [16], among others. Nonetheless, urine analysis and the finding of new methodologies for diagnosing abnormalities is still an area of great interest in the scientific community [17,18,19].

The currently available diagnostic methods for detecting UTIs in a hospital context are mainly laboratory methods requiring the pre-processing of samples, which include urine culture and urinalysis. Still, according to the European Association of Urology, the clinical diagnosis of UTI comprises onset or worsening of fever, rigours, altered mental status, malaise, or lethargy with no other identified cause, flank pain, costovertebral angle tenderness, acute haematuria, pelvic discomfort, and in those whose catheters have been removed, dysuria, urgent or frequent urination, and suprapubic pain or tenderness. In catheterised patients, pyuria, the presence of white blood cells in urine, is not diagnostic for CAUTI. Moreover, in terms of laboratory diagnosis, CAUTIs are defined by microbial growth of ≥103 cfu/mL of one or more bacterial species in a single catheter urine specimen or in a mid-stream voided urine specimen from a patient whose urethral, suprapubic, or condom catheter has been removed within the previous 48 h. In fact, the urine culture is the high goal standard for UTI diagnosis; it is the only method providing detailed information about the bacteria or fungal specimen causing the current infection. The main issue regarding this analysis is related to high costs and is time-consuming, requiring at least 24 to 72 h for the results to be available.

The limitations mentioned above have made urinalysis the most currently used diagnostic technique with a significant role in diagnosing and monitoring nephrological and urological illnesses [20]. This method is faster and cheaper than the urine culture and can also detect the presence of bacteria in the patient’s urine through a combination of physical, chemical, and microscopic analysis [12]. However, it requires no less than 24 h to get the results, thus delaying diagnosis and treatment for patients with CAUTI. Since there is no standardised protocol to conduct the urine analysis, the European Urinalysis Guidelines recommend a two-step strategy [21]:

- The first step involves a visual examination followed by the dipstick test. This test is used to exclude urine samples from further analysis if the parameters of haemoglobin, leukocyte esterase activity, nitrite, and protein are negative.
- In step two, if erythrocyturia, leukocyturia, bacteriuria or proteinuria are present, the urine samples are submitted for further analysis by microscopy. This step is crucial because the dipstick test screening by itself has low sensitivity, carrying the risk of missing infections and other urinary tract illnesses.

Urine dipsticks have many advantages, such as being quick, cheap, and functional in predicting UTIs in hospitalised patients. However, this method has some limitations. It is not a reusable test; the analysis result can be biased by the observer, and the degradation of the urine-reactive pads can occur. Moreover, its low sensitivity compared to urine microscopy and culture can put the screening accuracy of asymptomatic bacteriuria at risk [22]. False negative tests due to technical errors and excessively diluted, acidic urine or low pH may arise, and the nitrite test will be negative for bacteria that do not convert nitrate into nitrite. Any urine sample analysed less than 4 h after the last urination produces very inconsistent results [23] since it requires more than 4 h for bacteria to complete the nitrate-to-nitrite conversion. In sum, the first step in the urine analysis, as recommended in the European Urinalysis Guidelines, presents poor sensitivity and thus bears the risk of missing infections or other urinary diseases [24,25,26].

Hence, a more reliable and economical first-step approach to rule out urine infections is necessary. For such an approach to become successful, different requirements should be met:

- Its outcome must not rely on observer judgment;
- The ability to detect different types of microbial infections (bacteria or fungus) with equal sensitivity, even in the absence of nitrite;
- A reusable methodology that is more economically advantageous in the long term.

We thus propose a spectroscopic-based methodology combining fluorescence ratiometric and transmittance measurements as a first-step approach for urine analysis. To further reinforce the need for a new methodology for this end, we include, at the beginning of our results section, a survey made to Portuguese healthcare professionals to investigate how problematic CAUTI are and if a new methodology for urine monitoring would be welcome.

## 2. Materials and Methods

### 2.1. Surveys

A survey was conducted through the Google Forms to investigate the dimension of CAUTI in Portugal at health facilities in order to understand the routines with catheterised patients and determine which procedures are normally undertaken to monitor and detect UTI. This survey was only directed towards Portuguese healthcare professionals, including doctors, nurses, and nursing assistants, and all were invited to provide their concerns and suggestions about the matter. A total of 100 healthcare professionals replied. The anonymity of all participants was preserved.

The following questions were asked:

1. Hospital and department, health professional (doctor, nurse, or nursing assistant)?
2. Did you ever work daily with patients in continuous catheterisation for at least five days (yes or no)?
3. Have you witnessed any cases where patients have developed a urinary tract infection due to the catheter insertion (yes, no, or do not recall)?
4. If yes, how often (rarely, often, or very often)?
5. As a healthcare professional, what is the priority level of these infections (CAUTIs) in asymptomatic hospitalised patients regarding an early diagnosis and treatment (low priority, medium priority, or high priority)?
6. Which methods are used to detect signs of urinary tract infection in catheterised patients (thermometer, dipstick urine test, urine colour observation)?
7. During a regular work shift, how often is each of these methods used on catheterised patients who do not present any symptoms of urinary tract infection (none, once, twice, three or more times)?
8. Select the worst features of each method regarding effectiveness, hygienics, speed, user-friendliness, and reusability.
9. Would it be helpful to implement a new methodology for CAUTI early detection or just urine monitoring in the hospital routines (from the range of useless to extremely useful)?

### 2.2. Urine Samples

Urine samples for this research were provided by a large medical clinic. As a matter of protocol and logistical considerations, the urine samples were retrieved for this experimental activity approximately 24 h after being normally analysed by the clinic. According to the medical clinic analysis, each sample provided was labelled as a “Positive Sample” (urine sample with infection) or “Negative Sample” (urine sample with no infection). The medical clinic performed a complete urinalysis of each urine, including urine culture for samples that yielded positive white/red blood cells/nitrite assays and/or microscopic urinalysis. We studied all the urine samples on the same day they were retrieved from the medical clinic. Throughout this manuscript, urine samples identified with UTI will be referred to as “Positive Samples” and those that do not have infection as “Negative samples”.

Considering the motivation of this work, it was essential to use the urine samples in their original form (as they were collected), without submitting them to any dilution, centrifugation or other separation methods, to ensure that the methodology developed can be applied to situations where it is intended that sample manipulation is kept to a minimum.

**Figure 1:**
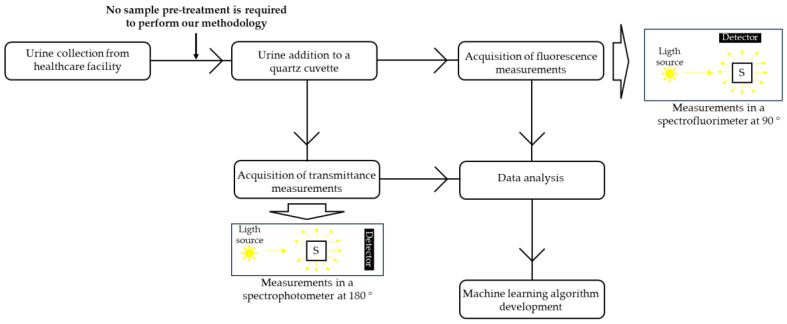
Schematics of the experimental setup followed in this work. In the scheme, “S” stands for sample compartment. In this scheme, one of the main differences between performing measurements in a spectrofluorimeter and in a spectrophotometer is highlighted. In a spectrofluorimeter, measurements are performed at a 90° angle between the light source and the detector, which means that only photons emitted at a 90° angle are detected. In a spectrophotometer, the measurements are performed at a 180° angle, which means that only light transmitted at a 180° angle will be detected. It is also important to emphasize that urine samples were studied directly with no sample pre-treatment, allowing for a quick sample analysis.

A total of 81 samples were used in this study, 40 positive and 41 negative. A scheme of the experimental setup followed in this work can be found in Figure 1.

### 2.3. Spectroscopic Measurements

As mentioned, to propose a reliable spectroscopic-based methodology as a first-step approach for urine analysis, we performed absorbance, transmittance, and steady-state fluorescence intensity measurements. All spectroscopic measurements were performed in 1 cm × 0.4 cm Hellma^®^ semi-Micro, Suprasil^®^ quartz fluorescence cuvettes. Samples were excited along the 1 cm pathway, and the emission was collected along the 0.4 cm pathway. Milli-Q water was used as a blank, but it presented only a marginal intensity at the wavelengths used, so the methodology proposed in the results and discussion section does not require the use of water or any other blank, which simplifies its implementation.

Absorbance and transmittance measurements were performed in a Jasco V-560 spectrophotometer (Easton, MD, USA). Before starting the measurements, the baseline was acquired with Milli-Q water. Spectra were acquired in the following conditions: 1 nm interval between two consecutive points, mean response, 1 nm bandwidth, and 400 nm/min of scan speed. Regarding transmittance, the value at 600 nm was retrieved from the obtained spectrum. A wavelength of 600 nm is normally used in microbiology to follow cell growth due to the optical properties of the bacterial/fungal culture growth media and to prevent cell degradation during the process.

On the other hand, Rayleigh scattering is kept to a minimum and light scattering measured is (mainly) due to the cells (microbial or human cells like leukocytes or hematocytes) present in the urine sample. Thus, high transmittance values suggest a low number of particles (cells) in suspension, whereas low transmittance values are indicative of a high number of particles in suspension. In addition to this, it is not expected that the other components of the urine will absorb at 600 nm.

Steady-state fluorescence measurements were performed in a Fluorolog-3 v2.2 spectrofluorometer (HORIBA Jobin Yvon, Edison, NJ, USA) with dual monochromators in excitation and emission in right-angle geometry, where the radiation source is a 450 W xenon arc lamp and the reference is a photodiode. The excitation and emission spectra were corrected using the correction files provided by the manufacturer. A water bath was coupled to the spectrofluorometer, enabling temperature control of the cuvette holder. In fluorescence spectroscopy, several conditions were tested in order to guarantee that the most sensitive parameters to urine composition were being used. For emission spectra, three excitation wavelengths were tested: 280, 290, and 360 nm. For excitation spectra, two emission wavelengths were tested: 410 and 520 nm. Finally, for obtaining the synchronous spectra, four wavelength offsets were explored = [30, 50, 70, 90] nm. In order to optimize sensitivity, the slits of the fluorimeter were also adjusted for each method above. Once the slits were defined, they were kept the same throughout all the samples. The number of counts per second was kept within the linear dynamic range. The light scattering was also measured with the fluorimeter at λex = λem = 600 nm.

### 2.4. Data Processing

After data acquisition, our purpose was to explore the significant parameters that enabled us to effectively distinguish positive urine samples (with infection) from negative ones (with no infection). The excitation, emission and synchronous spectra obtained for positive and negative samples were compared. As noted by Anwer et al. [27], we also observed that exciting a sample at λex = 290 nm could be a potential diagnostic tool to differentiate between normal urine and urine with bacteriuria. We further explored if this excitation wavelength was the one promoting the largest differences between negative and positive urine samples by comparing the emission spectra obtained in the 305 nm to 550 nm interval range for λex = 290 nm vs. λex = 280 nm. To develop an approach that would yield an instrument-independent output, a ratiometric methodology was considered, and for that purpose, the ratio (*R*) between the fluorescence intensity at the maximum emission peak (*M*) and 305 nm (*V*) was determined:

*R* = *M*/*V* (1)

The value of fluorescence intensity at 305 nm was chosen because the vast majority of the samples (whether negative or positive) showed, at this wavelength, identical fluorescence intensity. This data processing ensures that, independently of the equipment used to perform the fluorescence measurements, the output data can be compared using this ratio. The scattering signal measured at the fluorimeter for each sample is the average of seven acquisitions.

Five main parameters were extracted and explored in order to ascertain how they could be used to distinguish between negative and positive urine samples:

- The fluorescence intensity value at the spectrum maximum peak (*M*);
- The fluorescence intensity value at 305 nm (*V*);
- The ratio value between the M and V (*R*);
- The transmittance value at 600 nm (*T*);
- The light scattering averaged value.

Our unpaired dataset was analysed by a Mann–Whitney U test (non-parametric test) since the ratio between the variances was more than 3. The significance level of each parameter was analysed through the *p*-value, and for each statistically significant parameter, the range of values considering the confidence interval (CI) of 99% was determined to achieve the highest significant features to differentiate the urine samples into positive and negative.

### 2.5. Machine Learning

Following the statistical analysis, it was important to consider an autonomous classification process for the urine samples based on the extracted parameters. For this purpose, MATLAB R2020a (MathWorks, Natick, MA, USA) toolboxes were used. The Classification Learner app from MATLAB was used to select the most appropriate machine learning algorithm for the existing data. This app performs automated training to search for the best classification model type in order to classify the data and has the possibility to explore and supervise the machine learning algorithms using various classifiers, test which features better fit the model, select individual models or train a group of the same type, etc. Before starting any training process in the Classification Learner app, the dataset was split in half to construct training and testing sets. During the input and predictors selection, the model validation strategies are also chosen. This validation is used to tune hyperparameters and to better evaluate the performance of the model. For this work, the k-fold cross-validation was the most suitable. By default, the k-cross-validation in MATLAB is 5 (k = 5), and since there is no standard k value, the model performance was evaluated for k = [4–6]. The evaluation criteria for choosing the best classifier were based on the highest values of the Confusion Matrix accuracy and Area under Curve. The results were compared, and only the classifier that presented the best performance was selected. Additionally, during the training phase, hyperparameter optimisation was implemented to achieve the best performance possible.

## 3. Results and Discussion

In this work, besides presenting an alternative first-step methodology to distinguish urine with infection from healthy ones or just for systematic urine monitoring, we also present the results of a survey answered by Portuguese healthcare professionals.

### 3.1. Surveys

**Figure 2:**
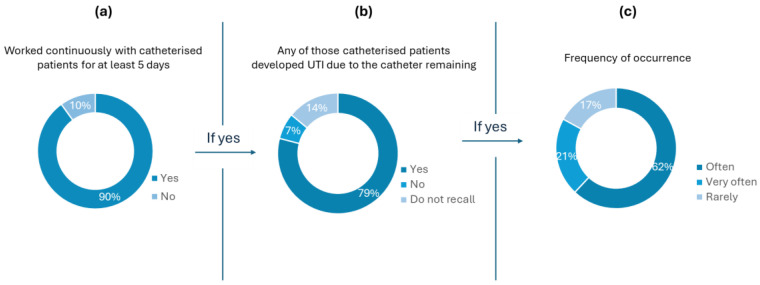
Results of questions 2 (**a**), 3 (**b**), and 4 (**c**) of the survey. The plot in (**a**) shows how many health professionals had already worked with catheterised patients. Questions 3 (**b**) and 4 (**b**) were targeted to the healthcare professionals who answered ”yes” to question 2 (**a**). The plot in (**b**) reports the results correlating to UTIs due to the catheter remaining in the hospitalised patient, and (**c**) presents how often it occurs.

Regarding the survey, most of the inquiries were from nurses (73%), then doctors (20%), and finally, nursing assistants (7%). Long-term catheterisation is considered when the catheter is inserted for one month or longer and is significantly linked to a high probability of bacteriuria. Conversely, short-term catheterisation is assumed to be less than a month. Taking into account the wide range of days (from 1 to 30) [28], it was important to establish a minimum, so the five-day minimum was determined. Thus, only health professionals who had worked with patients who had been catheterised for more than five days were considered for the next set of questions. According to Figure 2a, most of the interviewees (90%) had previously worked with catheterised patients for at least five days.

Figure 2b shows that 79% of the healthcare professionals who had worked with catheterised patients attributed the development of UTI to the catheter remaining. Moreover, only 17% of these professionals stated that it rarely happened (Figure 2c). The feedback to questions 2, 3, and 4 (Figure 2) shows that in the understanding of healthcare professionals, there is a strong correlation between the use of catheters and the development of UTI.

**Figure 3:**
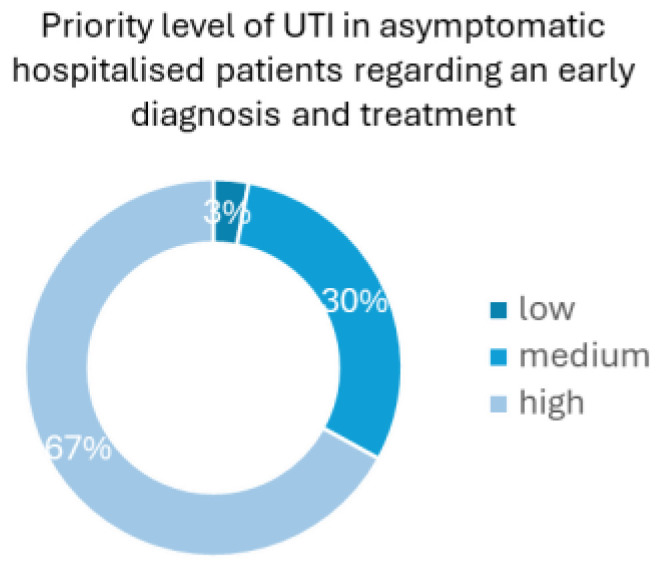
Priority level of how UTIs in catheterised patients are handled in Portuguese hospitals regarding early diagnosis and proper treatment (question 5): 67% of the health professionals considered a high level of priority, 30% and 3% considered a medium and low level of priority, respectively.

Regarding the priority level of an early diagnosis and treatment in asymptomatic catheterised patients, 67% of the health professionals considered it as a high priority, whereas 30% considered it a medium-level priority, and only 3% judged it to be a low-level priority health concern (Figure 3). The results presented in Figure 2 and Figure 3 reinforce the significance of the high need for UTI early detection in catheterised patients, an idea conveyed by the healthcare professionals themselves and not only derived by the numbers showing that UTIs are highly probable to develop in this type of patient.

**Figure 4:**
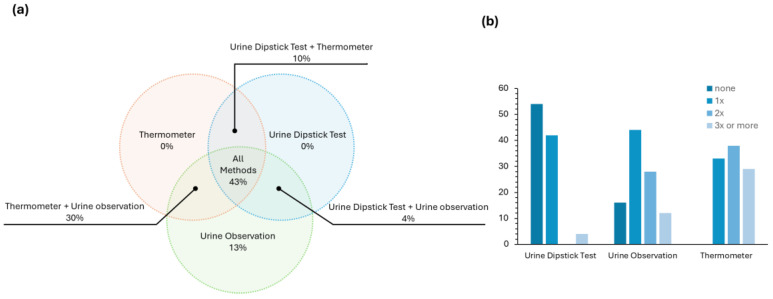
(**a**) The answers to question 6—the methods used to detect signs of urinary tract infection in catheterised patients (dipstick urine test, urine colour observation, and thermometer) are shown. (**b**) The answers to question 7 of the survey—during a regular work shift, how often each of these methods are used on catheterised patients who do not present any symptoms of urinary tract infection.

These first few questions of the survey (questions 2 to 5) validated what was already known from the literature—CAUTI is a health issue that needs to be tackled. Then, the survey explored which methods and diagnostic tools are used on a daily basis in Portuguese hospitals for an early and fast CAUTI diagnostic. The answers to this question (Figure 4a) revealed that in most cases, a combination of at least two approaches (either urine dipstick test + thermometer, urine dipstick test + urine observation or urine observation + thermometer) is employed. Many health professionals, ~43%, report using all three methods in a combined strategy for the evaluation of UTI. It is worth noting that the thermometer and the urine dipstick test are not used individually, which is in line with the accuracy issues addressed in the introduction concerning the dipstick test.

We were also interested in assessing how often each method was used individually in a normal work shift in patients without any symptoms of UTI. From the results presented in Figure 4b, most health professionals (~54%) do not use the urine dipstick test during their routine, and ~40% of them use it one time. The most used methodology is urine observation, most likely due to its simplicity.

**Figure 5:**
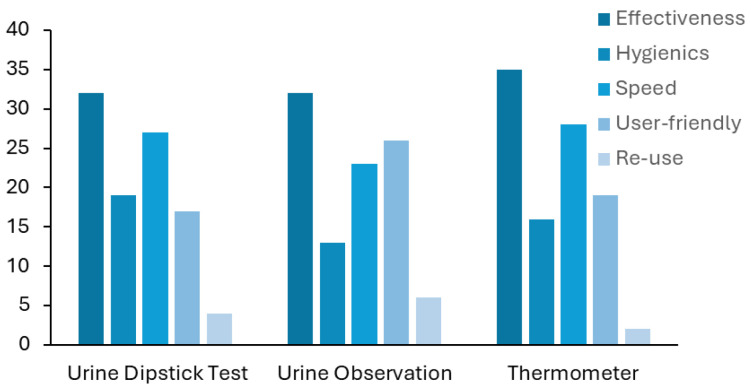
Answers to question 8, which regards the selection of the worst features. For each method—urine dipstick test, thermometer and urine observation—the worst utilisation characteristic was chosen among “Effectiveness”, “Hygienic”, diagnostic “Speed”, “User-Friendly”, and “Re-use”. Each interviewee could attribute more than one to each method.

Next, we asked the healthcare professionals what they considered to be the worst feature(s) of each method. They were able to choose between effectiveness, hygienics, diagnostic speed, user-friendliness, and re-use of each method. The results are displayed in Figure 5 and show that the healthcare professionals considered the lack of effectiveness to be the major drawback of all the methods. Despite not being pointed out by the health professionals in their answers, the reusability of the urine dipstick test is another major limitation of this test since each stripe can be used only one time.

**Figure 6:**
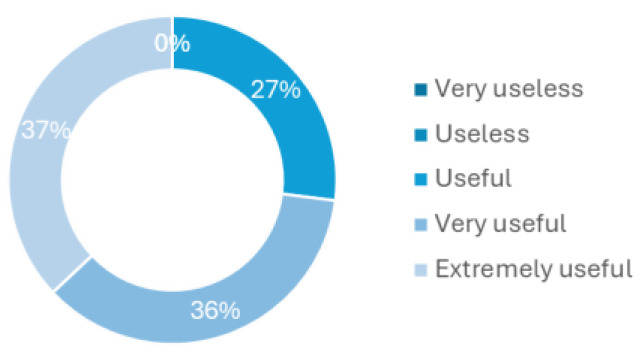
Utility level of a new methodology for early UTI detection or just urine monitoring.

The last question of this survey regards how a device for CAUTI early detection or just urine monitoring would be helpful to implement in the hospital routines. Figure 6 shows that all health professionals have considered it to be at least useful, with 36% believing it to be very useful and 27% extremely useful. This last question, in particular, was one of the main motivations for developing this new methodology to tackle the delayed diagnosis and treatment of CAUTI, especially in asymptomatic patients.

With this survey, the lack of reusable, economical, and reliable standardised methods for monitoring urine, especially in catheterised patients, for the early detection of UTIs became clear. These findings are also consistent with the literature, which is briefly explored in the introduction. For this reason, the present work aims to propose a new spectroscopic-based methodology combining fluorescence ratiometric measurements and UV–Vis absorption to measure sample transmittance as a first-step approach for urine analysis to screen for UTI.

### 3.2. Fluorescence Spectroscopy and Transmittance

It is a well-known fact that there are differences between the urine composition of a patient diagnosed with a UTI and that of a healthy patient [29,30]. We, thus, aim to explore these differences and develop a spectroscopic-based analysis to distinguish positive urine with infection from negative urine.

As one of the approaches, we employed steady-state fluorescence spectroscopy. Time-resolved measurements were also explored as an alternative; however, they cannot be applied to the purpose of this work mainly due to their lengthy acquisition time.

As already mentioned in the Materials and Methods section, fluorescence emission, excitation, and synchronous spectra were acquired. The fluorescence synchronous spectra with Δλ = 30, 50, 70, and 90 nm (Figure S1) did not allow for differentiation between positive and negative urine samples. The average synchronous spectra (Figure S1) obtained for positive and negative samples overlap almost completely and do not allow for a way to distinguish positive and negative samples.

**Figure 7:**
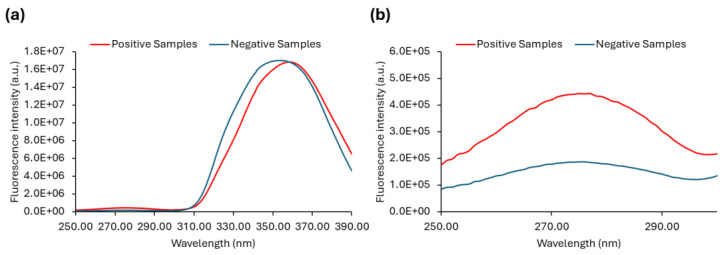
Intrinsic fluorescence of urine samples. (**a**) Average excitation spectra of autofluorescence of positive (red) and negative (blue) urine samples with emission wavelength set to 410 nm, and in (**b**), the range between 250 nm to 300 nm is highlighted. The slits were set to 7 nm, and spectra were acquired at 24 ± 1 °C.

We also analysed emission and excitation spectra. Since most fluorophores present in the urine have their excitation maxima in the UV range, we acquired excitation spectra with λem = 410 nm (Figure 7 and Figure S2). The excitation spectra of all the samples are shown in Figure S2, whereas in Figure 7 the average spectra are plotted. When analysing the overall positive and negative spectra (Figure S2), no noticeable differences were found between the two sets of samples. The average fluorescence excitation (Figure 7a) spectra practically overlapped and thus were not considered to be a valid approach to distinguish positive and negative urine samples. Still, if the excitation spectra are analysed in the range between 250 nm and 300 nm, spectral curves of lower intensity that are unnoticed in the plot in Figure 7 are present (Figure 7b). An excitation peak close to 280 nm is observed (Figure 7b), and a greater distinction between the fluorescence intensity of positive and negative urine samples is obtained compared to the maximum excitation peak centred at approximately 350 nm.

**Figure 8:**
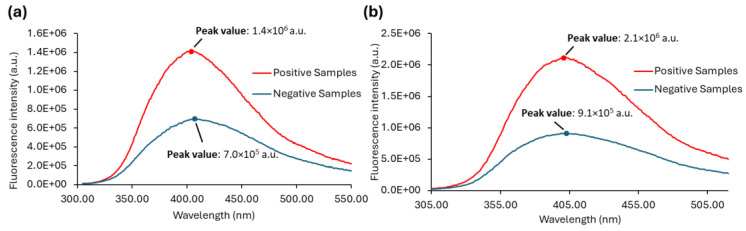
Average fluorescence emission spectra of positive (red) and negative (blue) urine samples when excited at (**a**) 290 nm and (**b**) 280 nm. The slits set for both emission spectra were 7 nm, and the data were acquired at 24 ± 1 °C.

As already mentioned, Anwer et al. [27] observed that at the 290 nm excitation wavelength, the negative urine samples exhibited a very low intensity of fluorescence emission when compared with the positive samples. We observed the same (Figure 8a and Figure S3) and extended our research to check that if, by exciting the samples at λex = 280 nm, we would improve the difference between positive and negative urine samples, either by selectively exciting fluorophores mostly present in positive samples or just by increasing their signal. Thus, the emission spectrum with excitation fixed at 280 nm was also obtained (Figure 8b and Figure S3) together with the emission spectrum with excitation fixed at 290 nm (Figure 8a) in order to investigate whether this approach would be a good way forward to be used as a diagnostic tool.

The emission spectra of all the samples with λex = 290 nm or λex = 280 nm are shown in Figure S3, while the average of the fluorescence emission spectra of positive and negative samples are shown in Figure 8. The maximum is also indicated for each set of samples.

The emission spectra of urine samples with λex = 290 nm (Figure S3a) and λex = 280 nm (Figure S3b) show the predominance of low fluorescence intensity values for the negative samples, whereas the positive samples display fluorescence intensities that are clearly higher. Negative urine samples can apparently be distinguished from positive ones by excitation both at 290 nm and 280 nm. The ability to differentiate between positive and negative urine is further demonstrated in Figure 8a,b, where the average spectra are shown. Although it is possible to successfully discriminate between positive and negative urine samples through excitation at both 290 nm and 280 nm, the fluorescence signal is stronger when the urine samples are excited at 280 nm (Figure 8b). The significant fluorescence intensity difference between positive and negative samples can be explored in order to differentiate positive from negative urine samples. Both the fluorescence intensity ratio between positive and negative samples and their absolute difference are higher in the case of λex = 280 nm. However, absolute maxima values cannot be used directly since they are instrument-dependent.

**Figure 9:**
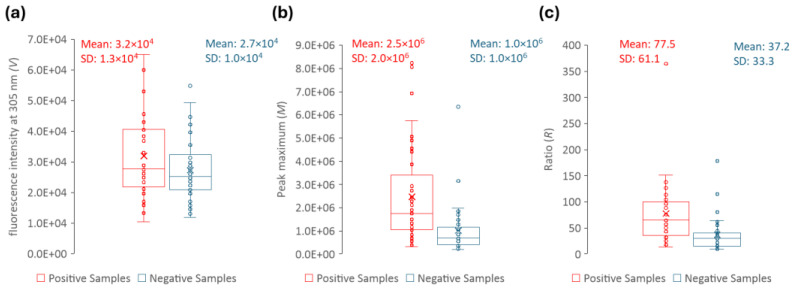
Box plots of (**a**) the fluorescence intensity at 305 nm (*V*), (**b**) the peak maximum (*M*), and (**c**) the Ratio (*R*) for both positive (red) and negative (blue) samples; the respective mean and standard deviation (SD) are shown. The parameters were extracted from the emission spectra with the excitation wavelength set to 280 nm.

In order to develop a reliable methodological approach, the ratio (*R*) between the peak maximum (*M*) and the fluorescence intensity at 305 nm (*V*) were determined for each sample (Figure 9). At 305 nm, all the samples have the same (or nearly the same) fluorescence intensity. This is shown in Figure 9a, where it becomes clear that, although there is some signal dispersion, the average fluorescence intensity at 305 nm is nearly identical for positive and negative urine samples. Moreover, its value is two orders of magnitude lower than at the peak maximum (Figure 9b). Thus, by using a ratiometric approach (*R*-value in Figure 9c) instead of a single wavelength measurement, our purpose is for the final intensity value to be more robust and more easily compared between different equipment and/or laboratories. Ratiometric methods are usually employed to study processes where spectral shifts are observed, which is not the case in the present work. However, the purpose here is to develop an approach capable of eliminating most instrument artefacts and intensity fluctuations.

We also tried to take advantage of the light-scattering ability of the samples and use it to differentiate positive from negative urine. To evaluate light scattering, we followed two different approaches: (1) light scattering in the spectrofluorometer was measured with λex = λem = 600 nm, and (2) the transmittance of the samples was measured in a spectrophotometer at 600 nm.

**Figure 10:**
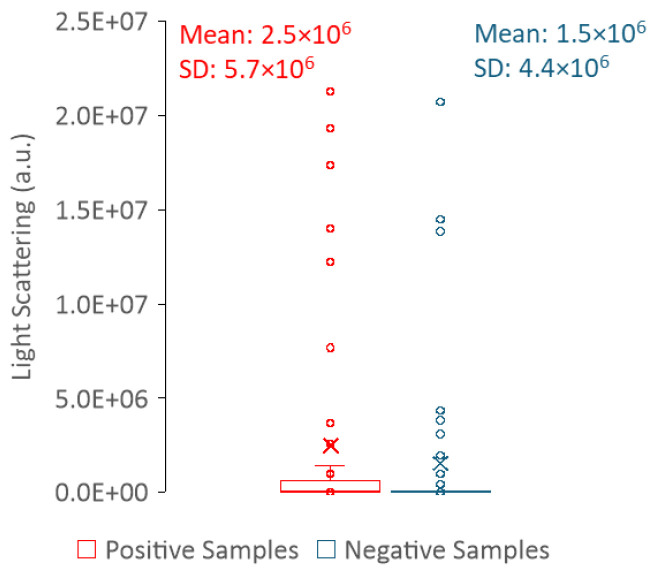
Box plots of the light scattering data for positive (red) and negative (blue) urine samples with the respective mean and standard deviation (SD). Data were acquired at 24 ± 1 °C, with λex = λem = 600 nm and slits set to 1.4 nm.

**Figure 11:**
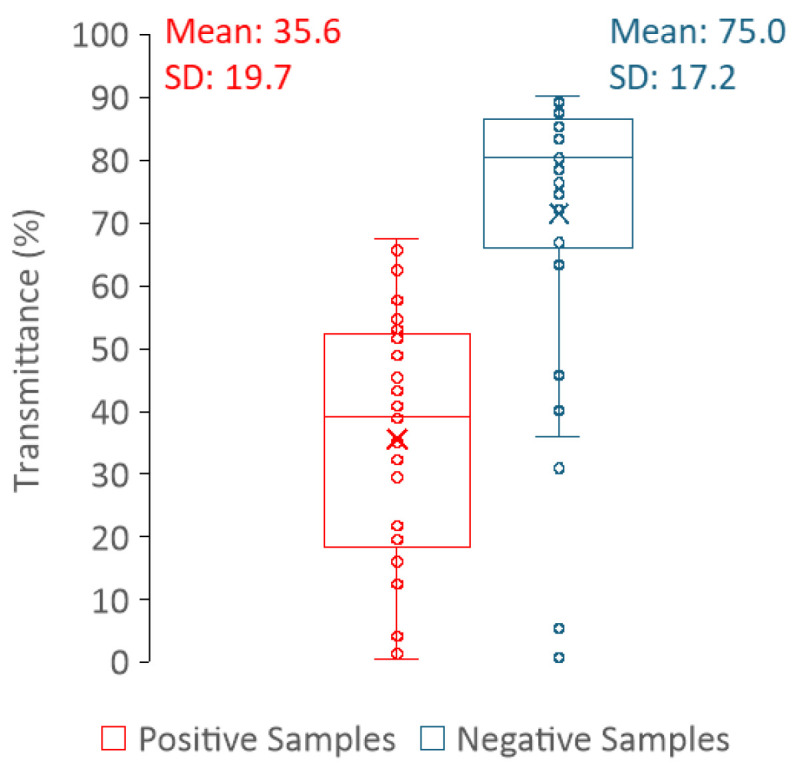
Box plots of the transmittance (*T*) at 600 nm for both positive (red) and negative (blue) samples with the respective mean and standard deviation (SD).

Regarding the light scattering of urine samples measured in the spectrofluorometer, it is hard to draw a correlation between the sample type (positive or negative) and the intensity value of the scattered light (Figure 10). Thus, measuring light scattering in a spectrofluorometer does not allow for a clear distinction between positive and negative samples. In contrast, the distribution of the transmittance values is clearly dependent on the sample type (positive or negative) (Figure 11), where high transmittance values correspond to negative urine samples, whereas low transmittance is indicative of positive urine samples. In fact, transmittance has been shown to be a strong tool to differentiate between positive and negative samples. This is because the predominance of cells or cell fragments in positive urine samples will scatter the light beam; thus, a smaller percentage of the incident radiation reaches the detector than in the negative samples. The fact that it is not possible to distinguish positive from negative urine using the light scattering measurements performed in the fluorimeter is most likely related to the geometry of the equipment. In the fluorimeter, light is collected at a 90-degree angle in relation to the light source; thus, only a fraction of light is analysed; in the spectrophotometer, light reaches the detector at a 180-degree angle in relation to the light source.

### 3.3. A New Spectroscopic Method to Detect UTI

For each sample, the fluorescence intensity value was retrieved at 305 nm (*V*), and the peak maximum of the fluorescence spectrum (*M*) and the transmittance (*T*) were retrieved. The ratio (*R*) between *M* and *V* was calculated for each sample. The distribution of the parameters that allow the distinction between positive and negative urine samples (*M*, *R*, and *T*) is shown in Figure 9 and Figure 11. These three parameters presented statistically significant differences between positive and negative samples with a *p*-value = < 0,001, shown in Table 1, where the descriptive statistics of each parameter are presented, including also the CIs of 99%. The data in Table 1 clearly show that *R*, *M* and *T* are effective parameters to distinguish the positive and negative samples. Moreover, between the *R* and *M* parameters, only *R* should be used to differentiate positive from negative urine since it is more robust and, as long as the instrument is internally calibrated, the data obtained in different instruments, days or laboratories/healthcare facilities can be directly compared if *R* is determined.

Envisaging the development of a methodology that can be used to distinguish positive urine from negative and taking into consideration the upper and low confidence intervals in Table 1 and data in Figure 9 and Figure 11, it was decided that:

For the parameter *R*—Concerning the positive samples, although the range of values is not narrow, only the lower limit of the CI is used for the method under consideration. In turn, in the negative samples, only the upper limit of the CI will be considered because the lower limit is not critical to be defined as long as it is greater than zero (Table 1).

For the parameter *T*—The distribution of positive and negative samples is such that the upper limit of the CI of positive samples and the lower limit of the CI of negative samples are still very distant. Thus, it was determined that only the upper limit of the CI was significant for the positive samples and the lower limit of the CI for the negative ones (Table 1). The lower limit of the positive samples can be defined as 0, and the upper limit of the negative samples can be defined as 100.

**Figure 12:**
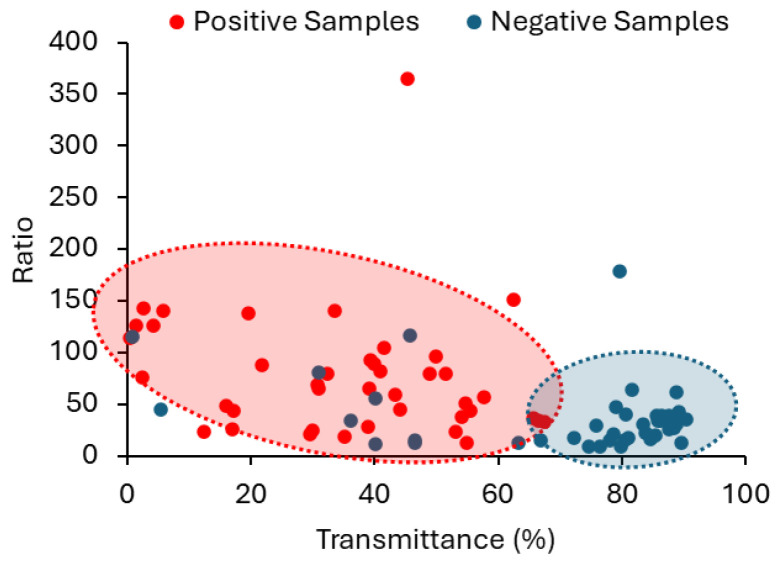
Scatter plot between the ratio value (*R*) and the transmittance (*T*) highlighting a clear separation between positive (red) and negative (blue) urine samples.

The suitability of employing a combined approach, using both *R* and *T* in the classification of urine samples, is well demonstrated in the scattering plot between *R* and *T* in Figure 12, where the two populations (positive and negative) are confined to different areas. Still, with the methodology we propose, it does not make sense to simply have a binary classification with just two class labels: Negative and Positive. This way, we suggest two different degrees of probability, depending on the CIs established for *R* and *T* parameters. If *R* and *T* obtained for a sample are within the CIs corresponding to the positive samples, the sample is classified as “high probability of a positive sample”. If *R* and *T* obtained for a sample are within the confidence intervals corresponding to the negative samples, the sample is classified as “high probability of a negative sample”. When only *R* or *T* fall within the established confidence interval for positive samples, the probability of a sample being positive (or negative) is lower. It is then classified as “Probability of a positive sample”. The approach currently recommended by the European Urinalysis Guidelines regarding the first step in urine analysis involves a visual examination followed by the dipstick test. The results of the dipstick test are available within 60 to 120 s. This is how long it would take to analyse each sample using our methodology. 

Similarly to the dipstick test, our approach does not require any pre-treatment of the urine samples, and the spectroscopic measurements are quickly obtained. Moreover, the dipstick test presents a sensitivity (true positive/(true positive + false negative) that does not exceed 80% and a specificity (true negative/(true negative + false positive)) of 60% [31]. It is a method that yields a high number of false positives. The output of the dipstick test is highly hindered if Gram-positive bacteria that do not convert nitrates into nitrites are the cause of infection. Different authors consider the dipstick test to be either a non-reliable assay in predicting UTI [32] or that its use to rule in an infection remains doubtful [26]. The microscopic urinalysis has a sensitivity of 91% and a specificity of 68%. 

On the other hand, the accuracy of our machine learning algorithm presents a promising value of 90.2%. Thus, we considered that our approach would be more reliable than the dipstick test in guiding the healthcare professional’s decision to send a urine sample to urine culture or not. We also believe that our approach would be considerably less expensive in the long term. 

To demonstrate, let us consider a hospital ward with 10 catheterised patients, and the dipstick test is performed twice a day on each of those patients, thus a total of 20 dipstick tests per day. Each stripe costs EUD 0.5 (hospitals and other large healthcare facilities might obtain more competitive prices since they acquire large quantities), which corresponds to an expense of EUD 10/day. This means that in 1 year, a total of EUD 3650 would have been invested for monitoring the urine of 10 patients. According to our calculations, our instrument should cost approximately EUD 20k. Thus, an initial investment of EUD 20k would be compensated in less than 6 years (the total investment in dipstick test stripes in 6 years would be EUD 21.9k), considering only 10 patients. Since the life expectancy of such instruments is close to 20 years, our approach is not only potentially more reliable but also more economically advantageous.

We have already tested our methodology and its capability to distinguish positive from negative urine. We have developed an interactive application for automatic UTI detection in MATLAB through its Design App. We tested different machine learning algorithms. We used *R* and *T* to train the models, and the best performance was obtained for the k-nearest neighbours machine learning algorithm with k = 5. As for the accuracy performance, although it is not a 100% accurate methodology, this model presented the highest value in the confusion matrix accuracy with 0.902 and an area under the curve with 0.750, which indicates a good-to-excellent capability to discriminate between the two defined groups using only two parameters, *R* and *T*. The interactive application is capable of doing an automated analysis of the fluorescence and transmittance spectra, calculating the value of *R*, selecting the value of *T* at 600 nm and returning the classification of the samples (high probability of a positive sample, high probability of a negative sample or probability of a positive sample) based on the k-nearest neighbours machine learning algorithm. Given these considerations, there is a high potential for identifying healthy and unhealthy urine from the methodology developed using biological samples without any further pre-treatment. This also indicates that this methodology has the potential to be developed as a simple and rapid diagnostic tool for UTIs.

Moreover, we have also designed a portable compact instrument capable of performing spectroscopic measurements both at a 90° and 180° angle between the light source and the detector that allows fluorescence ratiometric measurements and transmittance in the same instrument. The long-term purpose is to have our instrument used in hospitals, medical clinics or other healthcare facilities with catheterised or bedridden patients to perform the first step in urine monitoring. Both the interactive application and the instrument are going to be submitted for a patent.

Implementing a new urine monitoring methodology like the one developed in this work in a hospital setting involves several critical steps to ensure effectiveness, compliance, and patient safety. We already discussed a needs assessment by pointing out that our methodology has the potential to be a more reliable first-step approach for urine monitoring than the dipstick test without compromising test speed or patient comfort. Next, different stakeholders should be engaged. It is currently acknowledged that the delivery of healthcare is a multi-stakeholder activity and that the engagement of each stakeholder is pivotal for the successful development and service quality of a care pathway. A multidisciplinary team comprised of different healthcare providers (nurses, physicians), lab technicians, IT staff, and administrative leaders would have to be included in the implementation of this methodology. Moreover, vendors would have to be involved in the process as well. At the first instance, we would select a test unit like nephrology for a pilot test and develop protocols detailing operating procedures, including sample collection, analysis, reporting, and waste disposal. 

Personnel training is of pivotal importance for the successful implementation of our methodology. Comprehensive training sessions on how to use the system would be provided. Data interpretation by the healthcare professional is not necessary since our approach directly displays the probability level of infection, which is another advantage over the dipstick test. Patient compliance would not represent a problem since our methodology involves the same level of patient involvement as the dipstick test. The implementation would have to be in phases so that we could manage any challenges effectively. Another important aspect would be to ensure compatibility with the hospital’s electronic health record system for seamless data recording and analysis. A monitoring and evaluation step involving the tracking of key indicators and feedback collection from healthcare professionals is also foreseen. Finally, to promote the continuous improvement of the newly implemented methodology, any issues identified during evaluation would be addressed, and hospital protocols would be updated to reflect the new methodology.

## 4. Conclusions

The present work was motivated by the lack of commercial and medical urine monitoring solutions to help in early UTI diagnosis, particularly in catheterised patients. Thus, our main goal was to develop a methodology capable of distinguishing between healthy urine and urine with infection requiring only minimal sample handling.

The surveys conducted with different healthcare professionals were found to be highly important for the collection of real-life perspectives and evidence supporting the need for an on-early-stage detection of CAUTIs. The survey was restricted to Portuguese healthcare professionals, which happens to be the European country with the highest prevalence rate of HAIs (11.7%), with most of them related to UTIs. Portuguese healthcare professionals report that urinary catheterisation is a common procedure performed in hospitalised patients and that this procedure is highly correlated to UTIs. This strong correlation is on the basis of the professionals’ need for an early diagnostic tool for the timely treatment of CAUTIs, independent of whether the patient was admitted due to urinary tract-associated pathologies or not. In terms of pre-diagnostic methods and urine monitoring, there is no standardised methodology to answer this evident need. According to the surveys, the patient’s urine is subjectively and irregularly evaluated; it is up to the professional to decide how and when this evaluation should be conducted. Additionally, all three urine monitoring methods under consideration in the survey (urine dipstick test, thermometer, and urine observation) were deemed inefficient in detecting CAUTI, especially in asymptomatic patients.

In summary, the reply to the survey supports and reinforces the existing literature that conveys the importance of a standardised method for urine monitoring. In this work, we propose a method that efficiently distinguishes positive urine for infection from negative urine. To achieve this, several spectroscopic approaches were exploited throughout this work. Some of them were discarded due to their inability to differentiate positive from negative urine. Only three parameters revealed statistical significance between the two groups of samples: *M*, the peak maximum of the emission spectrum with λex = 280 nm; *R*, the ratiometric measurement between *M* and the intensity value at 305 nm; and *T*, the transmittance of the urine sample. Since it is intended to develop a methodology whose output can be directly compared between different laboratories or healthcare facilities, only the *R* and *T* parameters were considered for the method development. This is because only *R* and *T* ensure an instrument-independent measurement.

To the best of our knowledge, this is the first time that fluorescent ratiometric measurement and transmittance have been employed in the context of UTI detection, especially in a combined and complementary manner. The work and instrument developed have no intention of replacing a complete urinalysis or urine culture, which are high-quality exams to detect UTIs, but rather to create a monitoring method that can substitute the dipstick test as the first step in urinalysis to evaluate and alert the possible existence of UTI development, particularly in catheterised patients since they are the most affected by the lack of an early diagnosis.

## Figures and Tables

**Table 1:** Descriptive statistics for *M* (peak maximum of the fluorescence spectrum), *R* (ratio between the intensity values at *M* and 305 nm), *T* (transmittance), and respective confidence intervals (CIs) of 99%.

Sample		Mean	SD	Lower 99% CI	Upper 99% CI
*M*	Positive	2.47 × 10^6^	2.08 × 10^6^	1.59 × 10^6^	3.35 × 10^6^
	Negative	1.02 × 10^6^	1.09 × 10^6^	5.63 × 10^5^	1.47 × 10^6^
*R*	Positive	77.5	61.1	51.7	103
	Negative	37.2	33.3	23.4	51.1
*T*	Positive	35.6	19.7	27.3	43.9
	Negative	75	17.2	67.7	82.4

## Data Availability

The data presented in this study are available on request from the corresponding author.

## Supplementary Materials

The following supporting information can be downloaded at: https://www.mdpi.com/article/10.3390/s25020400/s1, Figure S1: Synchronous spectra with Δλ = 30 nm, 50 nm, 70 nm, and 90 nm of positive (red) and negative (blue) samples; Figure S2: Intrinsic fluorescence of urine samples; Figure S3: Intrinsic fluorescence emission spectra of positive (red) and negative (blue) urine samples when excited at (a) 290 nm and (b) 280 nm.
