# Supplementary material for: A Spectroscopic Methodology to Early Detection of Urinary Tract Infections
Source: Sensors (Basel). 2025 Jan 11;25(2):400. doi: 10.3390/s25020400 (PMC11768836; doi:10.3390/s25020400)
Supplement: Supplementary file 1 [file sensors-25-00400-s001.zip › sensors-3405072-supplementary.pdf]

Supplementary Material

A spectroscopic methodology to early detection of urinary tract infections

Mendes, Ana F.N.S.[1]; Matela, N.[1]; Coelho, João M.P.[1]; Marquês, Joaquim T.[2,*];

[1]Departamento de Física, Faculdade de Ciências, Universidade de Lisboa, 1749-016 Campo Grande, Portugal

[2]Centro de Química Estrutural, Institute of Molecular Sciences, Departamento de Química e Bioquímica, Faculdade de Ciências, Universidade de Lisboa, 1749-016 Campo Grande, Portugal

*** Correspondence:**

Corresponding Author

jmtmarques@ciencias.ulisboa.pt;

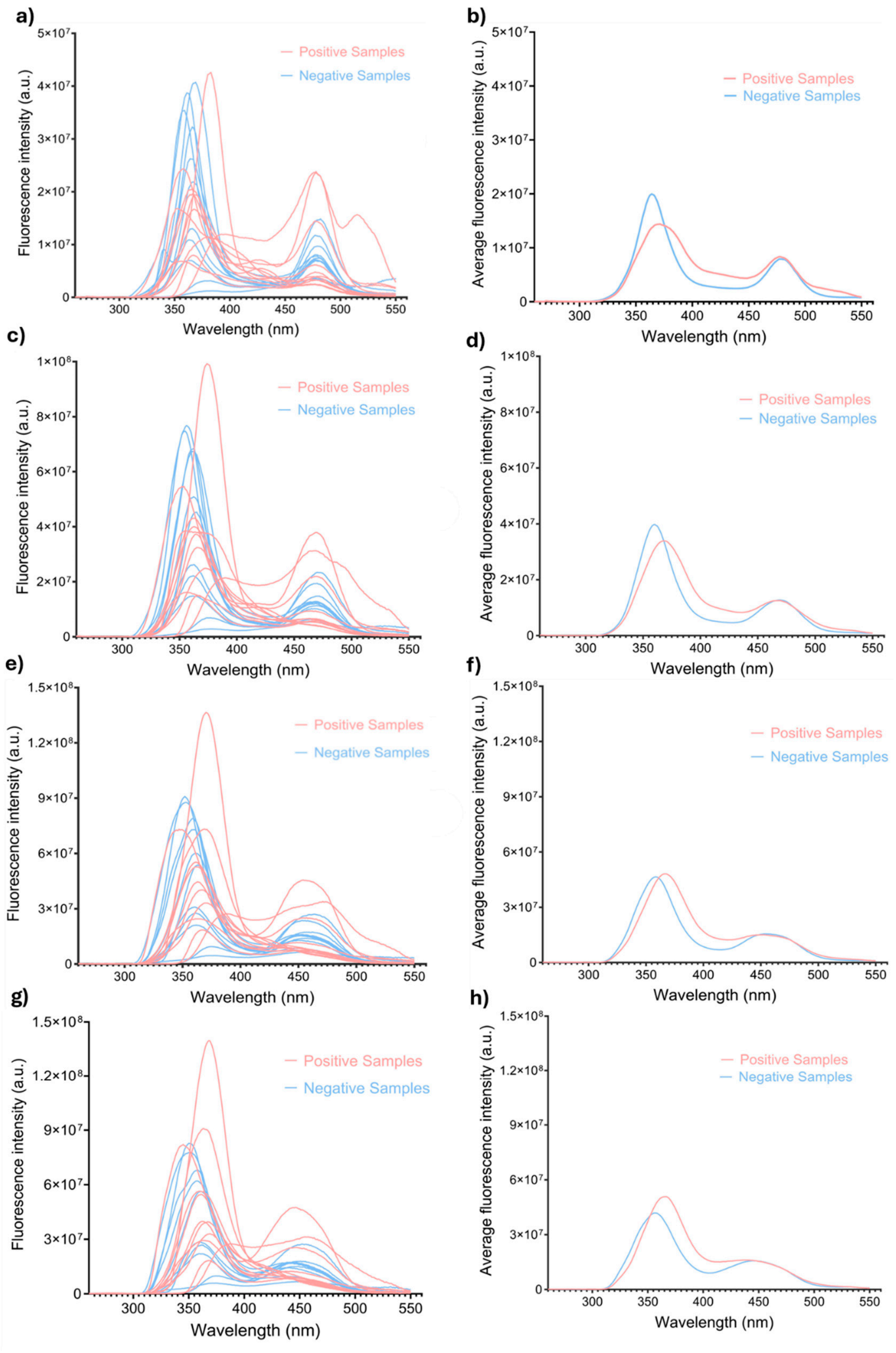

Figure S1 – Synchronous spectra with a) Δλ = 30 nm, c) Δλ = 50 nm, e) Δλ = 70 nm and g) Δλ = 90 nm of positive (red) and negative (blue) samples. The respective average spectra

are also shown in b), d), f) and h). The slits were set to 3 nm and spectra were acquired at 24 ± 1 °C.

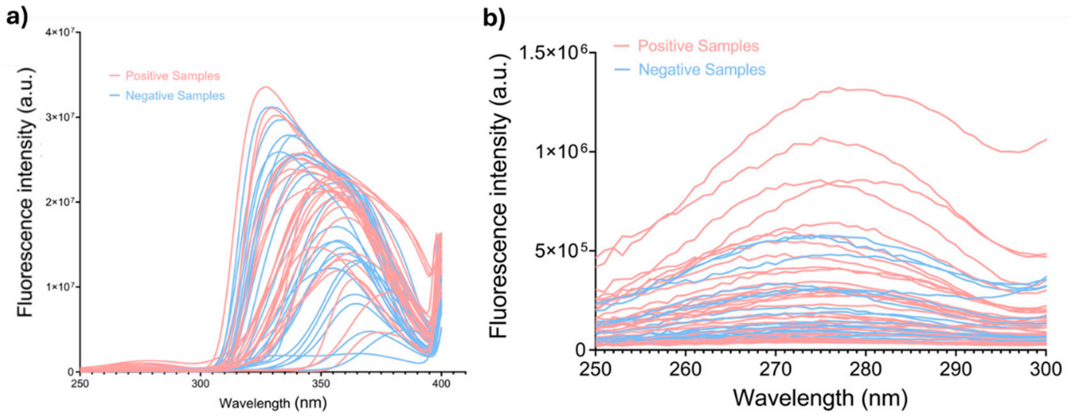

Figure S2 – Intrinsic fluorescence of urine samples. In a) the excitation spectra of all positive (red) and negative (blue) urine samples with emission wavelength set to 410 nm is shown. In b) the range between 250 nm to 300 nm is highlighted.

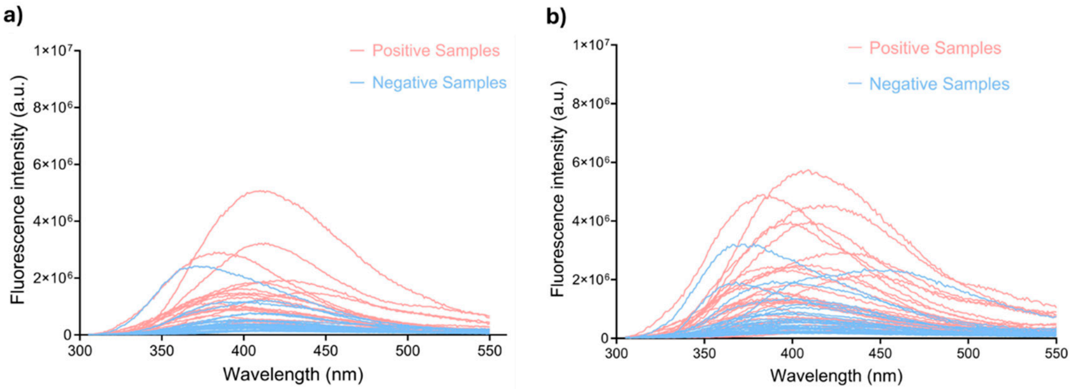

Figure S3 – Intrinsic fluorescence emission spectra of positive (red) and negative (blue) urine samples when excited at a) 290 nm and b) 280 nm.
